# Giant left ventricular pseudoaneurysm: a rare acute complication of radiofrequency catheter ablation for premature ventricular contraction

**DOI:** 10.1186/s13019-019-0946-3

**Published:** 2019-07-04

**Authors:** Hongxia Wang, Zhelan Zheng, Lei Yao, Yun Mou, Xiuqin Wang

**Affiliations:** Echocardiography and Vascular Ultrasound Center, The First Affiliated Hospital, College of Medicine, Zhejiang University, Hangzhou, China

**Keywords:** Pseudoaneurysm, Radiofrequency catheter ablation, Premature ventricular contraction

## Abstract

**Background:**

Radiofrequency catheter ablation is approved effective therapy for premature ventricular contraction. However, the rare but serious complication such as pseudoaneurysm should be given more attention. It is life-threatening due to the high risk of rupture. Only few cases have been reported in the literature. We herein report a huge acute left ventricular pseudoaneurysm after catheter ablation therapy.

**Case presentation:**

A 69-year-old man underwent radiofrequency catheter ablation for premature ventricular contraction at a local hospital. The patient developed shock the second day after ablation. A chest computed tomography (CT) scan showed pericardial effusion. Pericardiocentesis was performed, and the puncture fluid was a bloody pericardial effusion. The transthoracic echocardiogram revealed an 9- × 4-cm giant pseudoaneurysm with a cystic structure in the left ventricular inferior wall near the mitral annulus along the left atrium. The pseudoaneurysm was connected to the left ventricular cavity through a 8-mm neck, and the lumen was filled with systolic and diastolic blood flow. The patient underwent three-dimensional transesophageal echocardiography. The pseudoaneurysm and the tract was clearly visible. Emergency surgery was performed to resect the pseudoaneurysm. A bovine pericardial patch was placed on the neck of the pseudoaneurysm. Echocardiographic examination confirmed the absence of cardiac lesions after the operation.

**Conclusions:**

It is rare to see such a large pseudoaneurysm after radiofrequency catheter ablation. Clinicians should be allert to the potential risks to patients in the process of an effective treatment. Echocardiography plays an important role in the prompt diagnosis and prognosis of this disease. Emergency surgery is a better method for treatment of huge pseudoaneurysm.

**Electronic supplementary material:**

The online version of this article (10.1186/s13019-019-0946-3) contains supplementary material, which is available to authorized users.

## Background

Treatment of premature ventricular contraction includes drug and catheter ablation therapy. However, catheter ablation has become increasingly more important and has been widely used in recent years [1]. This technique is generally acceptable because of its high cure rate and low complication rate. Drug therapy is reportedly effective in only 25 to 50% of patients [2, 3], while radiofrequency catheter ablation is effective in up to 80 to 90% of patients with a recurrence rate of 10 to 15% [4, 5]. However, the rare but serious complication of ablation should be given more attention. We herein report a rare case of acute life -threatening left ventricular pseudoaneurysm after catheter ablation therapy.

## Case presentation

**Fig. 1:**
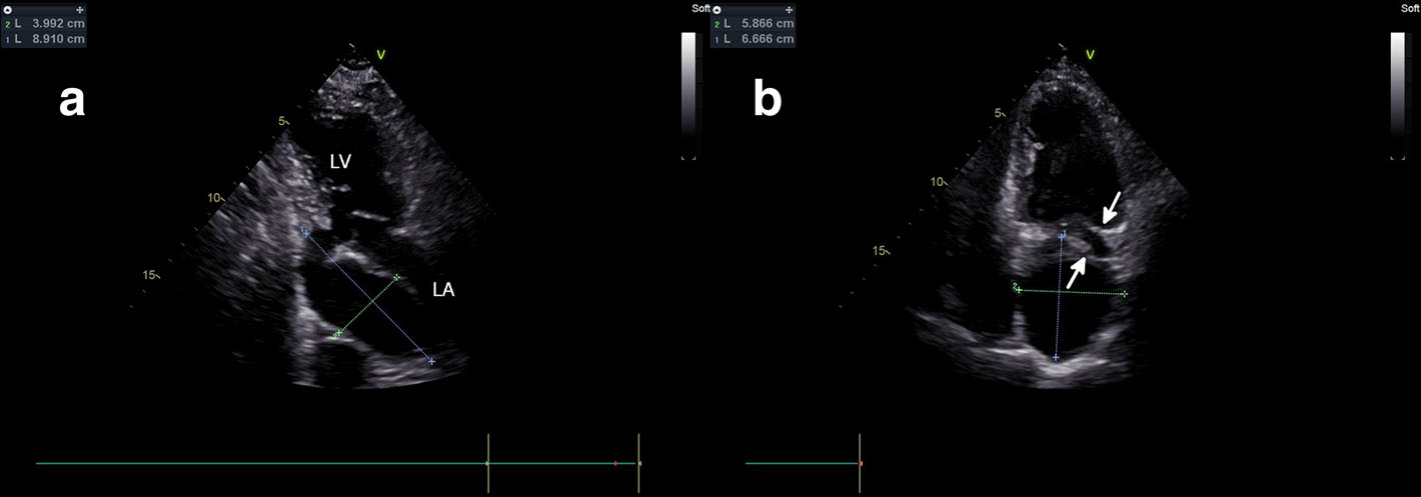
**a** Transthoracic echocardiographic apical 2-chamber view shows the left ventricular pseudoaneurysm(9x4cm) along the left atrium. LV (left ventricle), LA (left atrium). **b** Transthoracic apical 4-chamber view reveals the pseudoaneurysm(7x6cm), obviously compressed left atrium (white arrows)

**Fig. 2:**
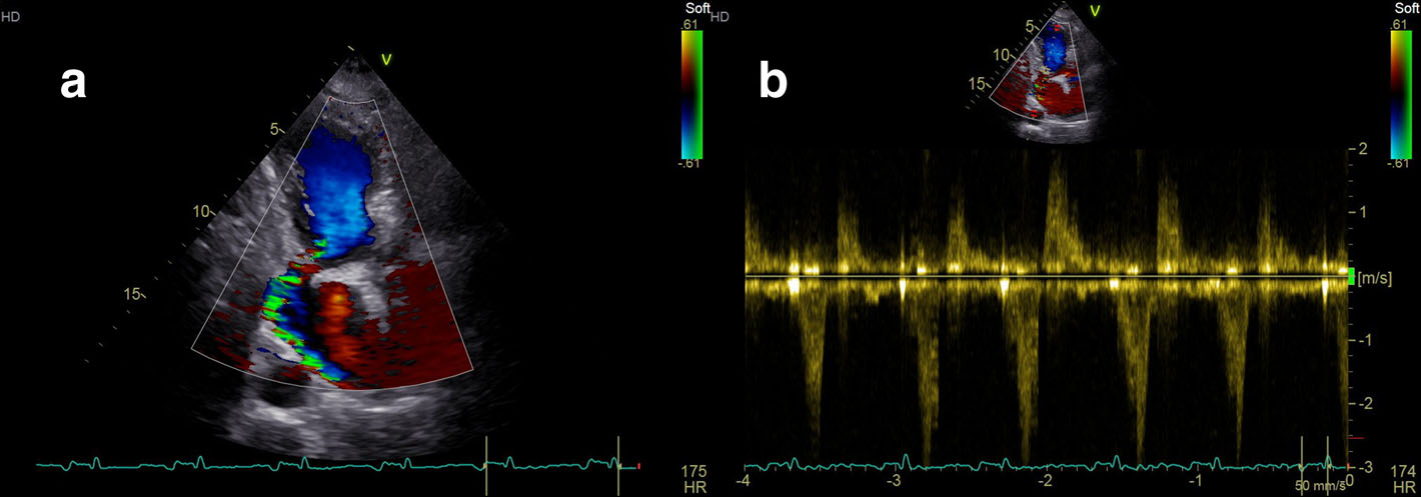
**a** Color Doppler imaging of transthoracic apical 3-chamber view displays the neck of the pseudoaneurysm near the mitral annulus. Systole frame shows blood flow from left ventricular into the pseudoaneurysm (blue flow). **b** Continuous wave doppler shows the systolic filling of pseudoaneurysm and the diastolic emptying into the left ventricular chamber (systolic velocity of 3.0 m/s and diastolic velocity of 1.7 m/s)

**Fig. 3:**
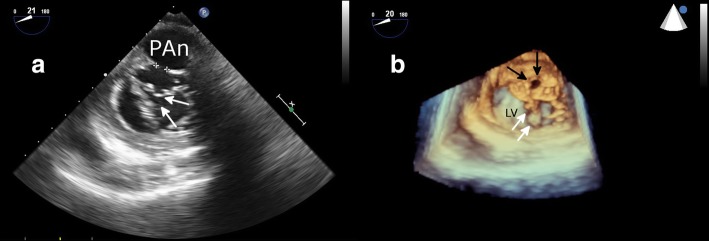
Transesophageal echocardiography. **a** Two-dimentional image clearly shows the plane struture of the pseudoaneurysm and the neck. **b** Three-dimensional image shows the neck shape (black arrow). PAn (pseudoaneurysm), LV (left ventricule), White arrow refers to the mitral valve

**Fig. 4:**
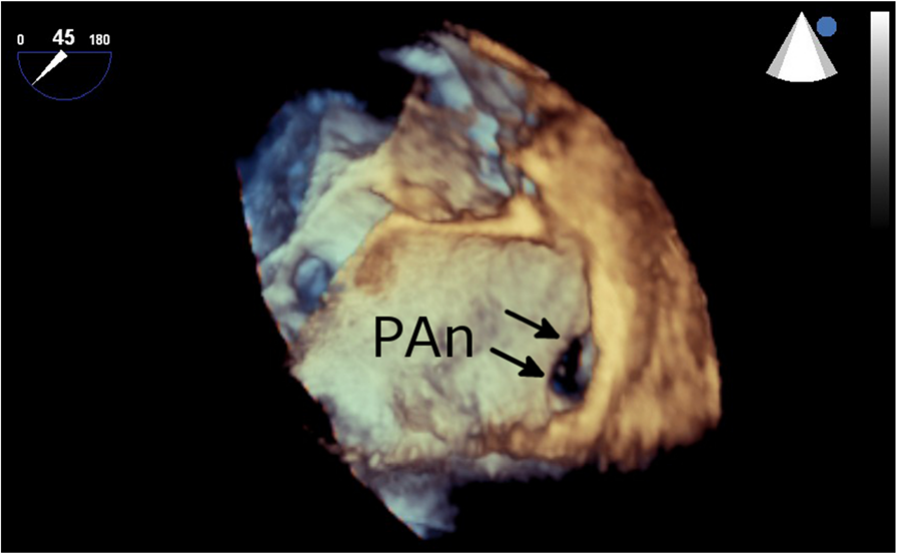
Three-dimensional transesophageal echocardiography reveals the pseudoaneurysm and the neck

A 69-year-old man underwent radiofrequency catheter ablation for recurrent symptomatic premature ventricular contraction at a local hospital. The patient had no history of coronary artery disease. An echocardiogram showed no obvious cardiac lesions before ablation. The patient’s earliest activation site of premature ventricular contraction was located in the left ventricular inferior wall. Ablation was performed to the endocardial portion with 30 W (temperature limit, 43 °C). The patient developed shock the second day after ablation. A chest computed tomography scan showed pericardial effusion. Pericardiocentesis was performed, and the puncture fluid was a bloody pericardial effusion. The patient’s symptoms were relieved, but complained of recurring dizziness and chest pain. The patient was transferred to our hospital for further diagnosis and treatment. Transthoracic echocardiogram revealed an 9- × 4-cm giant pseudoaneurysm with a cystic structure in the left ventricular inferior wall near the mitral annulus along the left atrium, which was obviously compressed (Fig. 1). The pseudoaneurysm was connected to the left ventricular cavity through a neck, and the lumen was filled with systolic and diastolic blood flow (Fig. 2) (An additional movie file shows this in more detail, see Additional file 1). In our hospital, the patient underwent three-dimensional transesophageal echocardiography. The pseudoaneurysm and the tract was clearly visible (Figs. 3 and 4), and the defect of the pseudoaneurysm was exactly at the point of radiofrequency energy delivery.

The pseudoaneurysm was surgically resected, and the defect was found in the inferior left ventricular wall near the posterior mitral leaflet. A bovine pericardial patch was placed on the neck of the pseudoaneurysm. The patient recovered well after the operation, and no premature ventricular contraction was observed.

## Discussion

Left ventricular pseudoaneurysm most often occurs after myocardial infarction and generally requires a surgical operation [6]. It may also rarely occur in association with cardiac surgery [7], trauma [8], and endocarditis [9]. Left ventricular pseudoaneurysm is a rare complication of radiofrequency catheter ablation, especially in the acute phase. Only two cases involving early complications have been reported. Dandamudi [10] described a 2.4- × 2.4-cm left ventricular pseudoaneurysm following premature ventricular contraction ablation of the endocardial portion of the left ventricular summit, and Gill [11] described a small pseudoaneurysm after radiofrequency ablation of supraventricular tachycardia. Three cases involving late complications have also been reported. Mansour [12] reported a case of a small left ventricular pseudoaneurysm near the mitral annulus as a late complication of low-energy direct current ablation. Koch [13] also reported a late large 6.1-cm left ventricular pseudoaneurysm following multiple endocardial and epicardial radiofrequency ablations for ventricular tachycardia. Auriau reported a 37- × 44-mm left ventricular pseudoaneurysm as a long-term complication of ablation of an accessory pathway. These cases reveal the rare life-threatening complications related to radiofrequency catheter ablation and the importance of prompt diagnosis for this potentially dangerous lesion.

The precise cause of left ventricular pseudoaneurysm is unclear. There are reports that high temperature can lead to perforation of the myocardium. This serious complication might be prevented by decreasing the power and reducing the operative time. The thickness of the left ventricular wall is not uniform; the thinner parts of the wall (such as the left ventricular summit) are more easily penetrated. Improvements in catheter ablation techniques will also help to avoid the occurrence of this serious complication.

Clinicians should be alert to the potential risks to patients in the process of an effective treatment. Several suggestions for clinical doctors are as follows. First, as noted above, left ventricular pseudoaneurysm is a rare potential serious complication of catheter ablation and should be given adequate attention. Second, cardiac rupture should be considered in patients with recurrent bloody pericardial effusion. Third, cardiac tamponade may also indicate the presence of a life-threatening complication. Finally, echocardiography plays an important role in the prompt diagnosis and prognosis of the disease. When symptoms are present, timely echocardiography is necessary, because this technique can promptly reveal effusion and lesions.

## Conclusions

This case reveals the importance of considering potentially life-threatening rare acute complications, such as left ventricular pseudoaneurysm, after radiofrequency catheter ablation especially in the acute phase. Echocardiography plays an important role in the prompt diagnosis and prognosis of the disease. Emergency surgery is an effective treatment for huge pseudoaneurysm.

## Additional file

Additional file 1: The movie file of color doppler imaging of the left ventricular pseudoaneurysm. Transthoracic apical 3-chamber view displays the neck of the pseudoaneurysm. Systolic frame shows blood flow from left ventricle into the pseudoaneurysm (blue flow), and diastolic frame shows blood flow from the pseudoaneurysm into left ventricle (red flow). (AVI 436 kb)

## Data Availability

Not applicable.

## Abbreviation

CT: Computed tomography
